# Modeling the biogeochemistry of sulfur in beech (*Fagus sylvatica* L.) stands of the Vienna Woods

**DOI:** 10.1007/s40808-020-00770-5

**Published:** 2020-05-12

**Authors:** Klaus Dolschak, Torsten W. Berger

**Affiliations:** Department of Forest- and Soil Sciences, Institute of Forest Ecology, University of Natural Resources and Life Sciences (BOKU), Peter Jordan-Straße 82, 1190 Vienna, Austria

**Keywords:** Sulfur deposition, Occult deposition, Sulfur biogeochemistry, SO_4_ soil solution, Nutrient balance model, Simulated annealing

## Abstract

In this article, we describe the setup and the application of a novel Sulfur Dynamics Model (SDM), with the aim to identify and quantify processes, which are crucial for the understanding of the biogeochemical sulfur (S) cycle of forest ecosystems. The simulator takes into account abiotic processes as well as biotic interactions between atmosphere, plant and soil. We applied the model to two Austrian beech stands where deposition of S and soil solution chemistry were monitored intensively over a two-year period. Under consideration of high historic loads and the more recent recession of atmospheric S deposition, we found a suitable model configuration where it was possible to assign both intra-annual fluctuations of the SO_4_-S in soil solution and long-time trends in the stream discharge to specific S transformation processes. We identified the interplay of microbial immobilization (the microbial conversion of solute sulfate to organic soil S) and mineralization as key driver of short-term fluctuations in the soil solution. In the long term, the delayed release of historically accumulated S is driven mainly by the slow mineralization of S rich plant biomass, recalcitrant to decomposition. Adsorption and desorption processes seem to play only a negligible role on our investigated stands. We conclude that our proposed model which is based on the current understanding of S biogeochemistry is sufficient to describe S dynamics on the investigated forest stands. The code file (SAS) for all model functions will be provided by the authors after request.

## Introduction

During the Twentieth century, Central European forest ecosystems received high loads of atmospheric S. Several reasons led to a sharp decrease in S emissions and accordingly deposition, starting in the late 1980s. From 1990 to 2016, sulfur emissions could be reduced by 81% in Austria (Umweltbundesamt 2018). This decrease was assignable to policy-driven shifts in the energy sector, mainly the reduction in the use of S-rich fossil fuel, coupled with the installation of end-of-pipe technology. With declining emission, also the deposition load to forest ecosystems decreased. In the period from 1984 to 2013 throughfall (plus stemflow) fluxes for an Austrian beech stand sank from 2.3 to 0.6 g m^−2^ year^−1^ (Berger and Muras 2016). However, the observed drop was only poorly reflected in the catchment output of most forest sites (Alewell 2001; Alewell et al. 2001; Pannatier et al. 2011; Prechtel et al. 2001). Similar patterns were also reported from Northern America: The observed decline of acid deposition was, in most cases, not accompanied with a decline of acidifying agents in the stream output of the investigated catchments (Watmough et al. 2005). This was in strong contrast to the prediction of, at that time, state-of-the-art ecosystem simulators, which forecasted a relatively fast response of solution and stream chemistry after a change in the deposition regime. Likens et al. (2002) postulated 4 mechanisms, explaining a negative input/output balance of S: (1) the release of S by bedrock weathering, (2) a net release from the ion adsorber, (3) the excess mineralization of historically accumulated organic S or (4) the underestimation of dry S deposition. Studies of the isotopic composition of the atmospheric S input and the catchment S output clearly pointed toward an organic source (Novák et al. 2000). Especially for catchments which received high historic loads of anthropogenic S, the isotopic composition of the stream discharge showed higher portions of ^32^S than the atmospheric input. Plants and soil microflora generally prefer the lighter ^32^S isotope toward the heavier, less abundant ^34^S (Mitchell et al. 2001; Zhang et al. 1998). Sulfur, which was biologically incorporated into organic material during phases of high deposition, was now being released steadily, in form of SO_4_-S by mineralization. Novák et al. (2000) state that a considerable amount of the atmospherically deposed S is cycled through the biosphere before being given off to the soil solution and the stream discharge. Ecosystem models such as MAGIC, SMART, SAFE or CHESS describe S storage and release based solely on sorption isotherms (compare Alewell 2001). They share their neglect in the possibility of biotic S cycling and the storage of S incorporated in an organic pool. In an attempt to model the stream chemistry of the output of a forested catchment in Germany with the MAGIC model, it was necessary to introduce an additional S source (968 mg m^−2^ yr^−1^) to match the simulated with the observed stream discharge (Prechtel et al. 2003). Gbondo-Tugbawa et al. (2001) found that the introduction of a plant S uptake and a mineralization module considerably increased the performance of the PnET-BGC model in describing the SO_4_-S output of a forested catchment.

In this work, we introduce a novel biogeochemical model to describe S dynamics of temperate deciduous forest stands. Besides the assessment of geochemical dynamics, we lay emphasis on biotic interactions: In addition to plant uptake, litterfall and the release of S via mineralization of plant organic material, we try to assess microbial immobilization (the microbial conversion of soil solution sulfate to organically bond S) in the forest soil as a crucial part of the biogeochemical S cycle. The model is applied to two beech stands in eastern Austria where deposition and solution chemistry were monitored closely over a two-year period. In this work, we try to answer the following questions:

- Is it possible to derive a model configuration that delivers a plausible representation of the current state of the investigated sites and of the observed temporal pattern in the soil solution?
- Does this model configuration provide insight, which biogeochemical processes are the key drivers of the observed intra-annual pattern in the SO_4_-S soil solution?
- Furthermore, is the simulator, which is calibrated on data from a very narrow timeframe, capable to provide a reasonable perspective regarding the future development of the stands’ S budget and balance? How long does it take for the stream discharge of S to decline to a pre-industrial level? Is a new steady state (balance between S input and S output) foreseeable within the model timeframe?

## Materials and methods

### Study sites

**Fig. 1:**
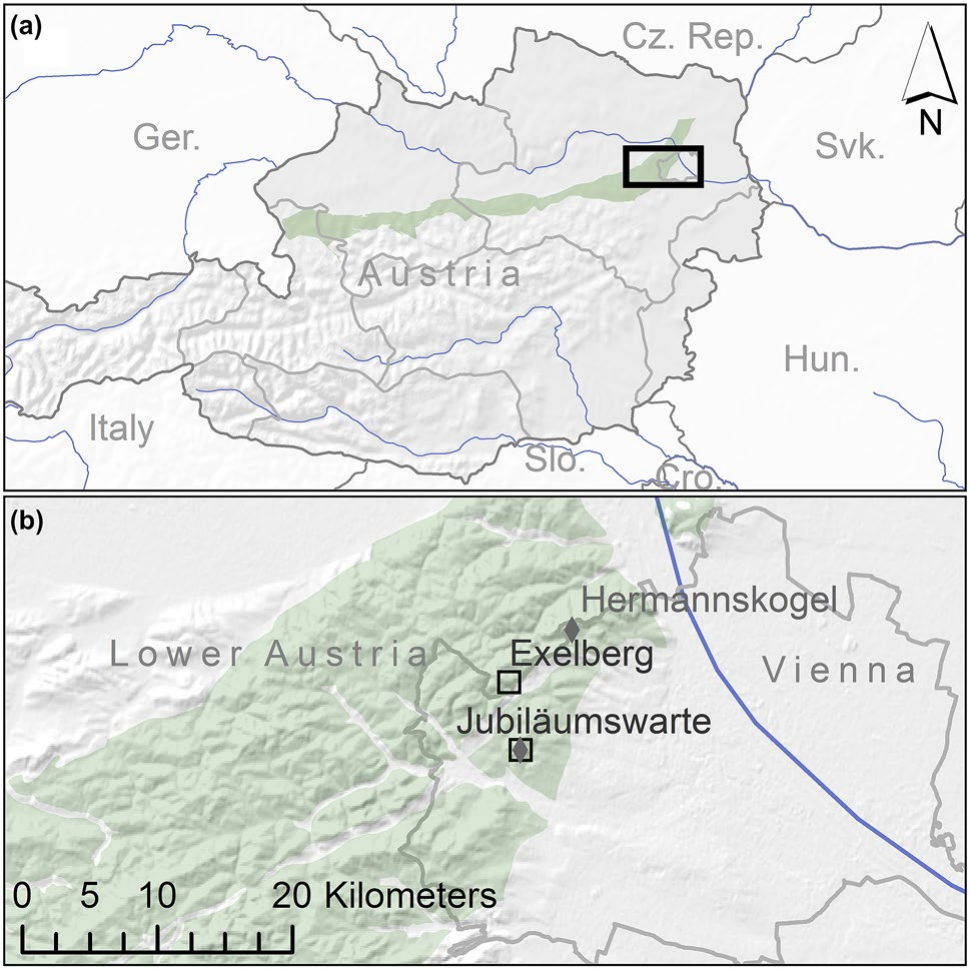
**a** Location of the investigated sites in Lower Austria and Vienna. Both sites share the bedrock Flysch (darker band area, crossing Northern Austria) and several other site characteristics. **b** Both forest stands (squares) are located at upper hill slopes, close to the ridge, facing SE. Due to the sites’ microrelief, in combination with the immediate proximity to the urban area of Vienna, they are deemed very susceptible for pollution-enriched air flow from south to easterly directions. Weather information and SO_2_ data (diamonds) were obtained mainly from Jubiläumswarte and Hermannskogel

The investigated forest stands are located in the northeasterly part of Austria at the eastern edge of the Vienna Woods (see Fig. 1). The parent material for soil formation is Flysch, which consists of old tertiary and mesozoic sandstones and clayey marls of maritime origin. Due to a high clay content, the saturated hydraulic conductivity is low, leading to frequent episodes of waterlogging. Therefore, the soil type is classified as Stagnic Cambisol according to the WRB soil classification (IUSS Working Group 2006), throughout both studied sites. The mean annual temperature in the study area is approximately 9 °C. The average annual precipitation is 660 mm (Wien–Hohe Warte).

The first site Exelberg (E) is located in Lower Austria close to the border to Vienna (48°14′40″ N, 16°15′18″ E), at an elevation of 460 m a. s. l. This site is a pure beech stand. We estimated its age to approximately 100 years. The stand is facing SE with an inclination of 22%. The second site Jubiläumswarte (J) is located within the municipal area of Vienna at the eastern edge of the Vienna Woods (48°13′12″ N, 16°15′56″ E), 2.8 km southeast of E, at an elevation of 440 m a. s. l. The site, which is also a pure beech stand with an estimated age of 125–150 years, is also facing SE, with an inclination of 15%. As a matured stand, it is showing signs of collapse but also strong natural regeneration. In contrast to the E stand, the bedrock of the J site contains calcareous material, reflected in higher base saturation and soil pH. Both forest stands are located at upper hill slopes, close to the hilltop. Due to their southeasterly exposition they are very susceptible to direct air flow, coming from the urban area of Vienna. Further details about forest site, stand and soil characteristics are given in Hanousek et al. (2017).

### Data sources

The simulator, which we outline in this work, runs on a daily timestep. Therefore, all input data must be provided in daily resolution. Sulfur enters and leaves the soil system mainly in aqueous solute form. To describe the short-term dynamics of S in the soil, we simulate water balance and water fluxes, using the formulation of the Water Balance Model (WBM) presented in the supplementary of Dolschak et al. (2019). We ran the WBM using meteorological input data from the weather station Jubiläumswarte (100 m distant from J) obtained from the Zentralantalt für Meteorologie und Geodynamik (ZAMG). The meteorological record of the mentioned station starts in 2011. For the timeframe before that year, we created a synthetic weather time series using neighboring stations with longer records; outside the timeframe with measured data, we applied a weather generator to create a synthetic record. We used the daily mean air temperature and snapshot measurements of soil temperature to generate a continuous record of soil temperature. The approach plus its parameterization is described in Dolschak et al. (2015). To determine the timing of the stands’ phenological key events, we used the phenological module, which is also presented in Dolschak et al. (2019).

Both investigated sites were monitored from April 2010 to June 2012 in a biweekly interval. Precipitation water was collected in the open field (bulk precipitation), as well as after the passage through the canopy (throughfall). A funneling apparatus was installed on one tree per site to collect stemflow. Soil solution lysimeters were installed at 10, 30 and 50 cm soil depth, each in 5 replications per site. Snapshot measurements of soil moisture (TDR-Trase) and soil temperature were taken in the course of the biweekly sample collection. Water samples (precipitation and soil solution) were analyzed for SO_4_-S content via ion chromatography (Dionex DX 500, USA).

**Fig. 2:**
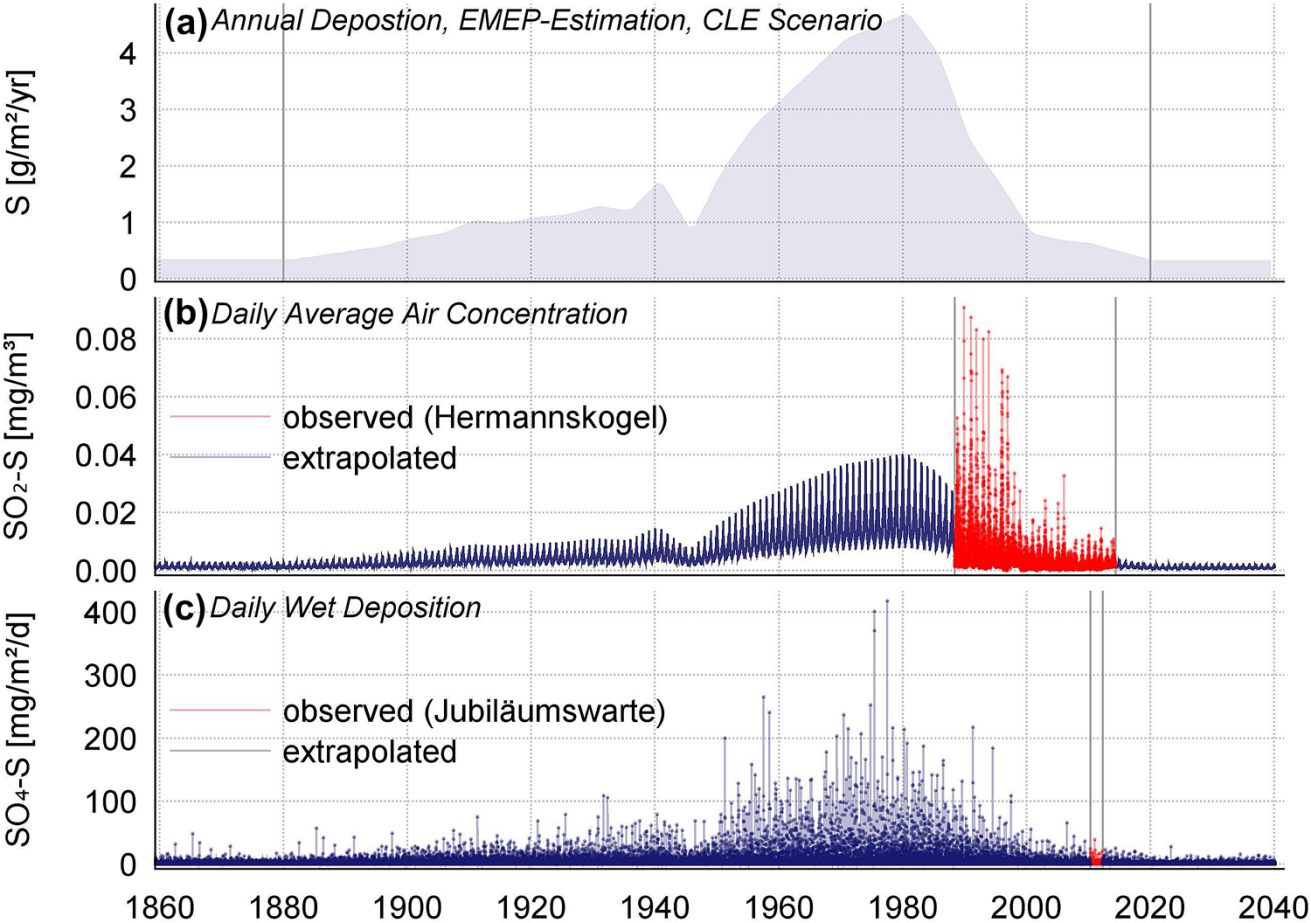
Extrapolation of observed deposition data to the model timeframe. The model timeframe spans from 1770 to 2100. Observations of S deposition and air concentration were available only for the very recent past. **a** To extend our input data time series beyond the observed time frames we used a dataset of SO_4_-S deposition estimates (EMEP), generated by Schöpp et al. (2003). The data estimates range back to 1880. The set also comprises estimates for the near future. For our purposes, we chose the Current Legislation Emission (CLE) scenario. Outside the defined timeframe, we extended the time series, assuming steady conditions. **b** Daily mean values of the aerial SO_2_ concentration for the site Hermannskogel, were available, ranging back to the year 1988. Due to the fact that SO_2_ concentrations exhibit a strong seasonal pattern, extrapolation was performed by calculating average values on a Day of Year base, which were then extended beyond the available timeframe. Here, the normalized EMEP time series served as a multiplier to scale the aerial concentration to historic/future conditions. **c** For daily wet deposition, we applied an approach, similar to the extension of the aerial concentration. Annual deposition sums where partitioned proportionally to daily precipitation events

Daily mean values of the aerial SO_2_ concentration were obtained from the Umweltbundesamt (Wiener Umweltschutzabteilung—MA 22 Luftmessnetz), for the site Hermannskogel, ranging back to the year 1988. To extrapolate these data to the pre-industrial era, we used a time series of the estimated SO_4_-S deposition, derived by Schöpp et al. (2003). This time series was normalized to a value of one for the period with existing data (compare Fig. 2b). Outside the timeframe, we multiplied the normalized estimate with the Day of Year (DoY) mean from the measured period. The data estimates range back to 1880. The dataset also comprises estimates for the near future. For our purposes, we chose the CLE (Current Legislation Emission) scenario. Outside the defined timeframe, we extended the time series, assuming static conditions before the first and after the last year of prognosis.

On both sites, bulk precipitation deposition of SO_4_-S (open field) was sampled from April 2010 to June 2012 in a biweekly interval. To achieve a daily resolution, we prorated the collected amount proportionally to the measured amount of daily precipitation. For the extension of deposition to the time period before measured data were available, we used a similar approach as with the SO_2_ air concentration (Fig. 2c).

### Model description

Disregarding S inputs from dry deposition and gaseous losses, S enters and leaves the undisturbed forest ecosystem mainly in aqueous solute form SO_4_^2−^ (Likens et al. 2002). Consequently, there is a strong link between biogeochemical S balance and the hydrologic balance. Also the S uptake by plants, the mineralization of S-containing biomass (Moyano et al. 2012; Sierra et al. 2015) as well as other microbially mediated S transformations in the soil are assumed to show a strong response to soil moisture (Aulakh et al. 2002; Janzen and Bettany 1987; Solberg et al. 2005). Therefore, an expedient description of the water balance seems crucial for modeling the S dynamics of a forest stand.

The simulator, which we outline in this article, works as an extension to the WBM; running the Sulfur Dynamics Model (SDM) requires the output of the WBM. Additional to that, the simulator requires daily information about SO_4_-S inputs via wet deposition above the canopy. To calculate the amount of dry and occult deposition, the daily mean SO_2_ air concentration is required. For the calculation of biological S transformations in the soil, the mean daily soil temperature of the soil is required. All other required input data are derived from the output of the WBM simulation. Fluxes, associated with the solute phase of S (canopy interception, snow dynamics, infiltration, bypass flow, percolation), are fully controlled by the associated water fluxes, defined through the WBM. All these processes are calculated, using the following scheme. The change in the content of the solute (mg/m^2^) of the desired pool is defined as the product of content and the relative change of the water content of the pool during the timestep.

1 $$\frac{\textit{dCont}}{{{\textit{d}}t}} = {\textit{Cont}}\frac{{\frac{\textit{dWater}}{{{\textit{d}}t}}}}{\textit{Water}}$$

**Fig. 3:**
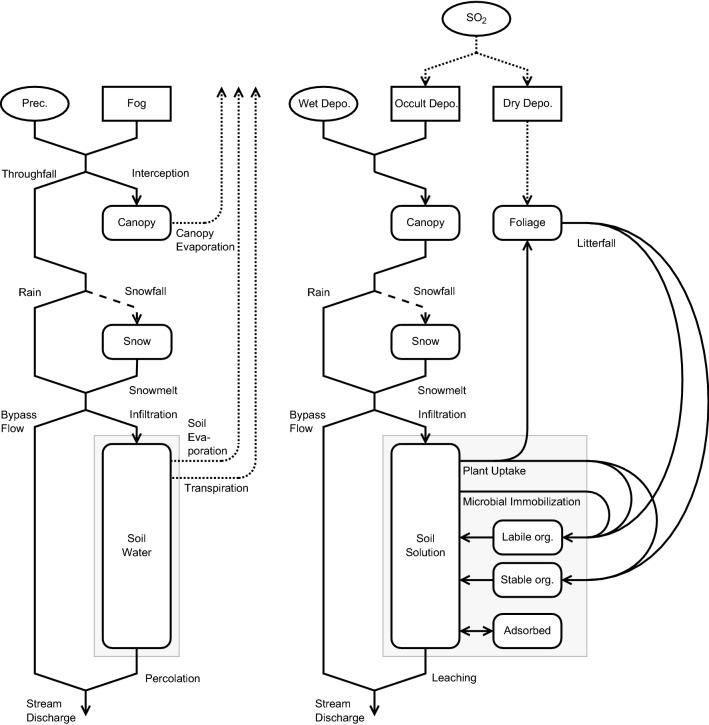
Flowchart of the Water Balance Model (WBM, left) and the Sulfur Dynamics Model (SDM, right). A comprehensive description of the WBM is given in Dolschak et al. (2019). The WBM states the hydrologic framework for the SDM. All S fluxes, associated with the solute phase, are controlled by the WBM

Analog to the WBM, the SDM can be divided into an aboveground and a belowground module. A flowchart of both formulations is given in Fig. 3. Only two aboveground processes are explicitly modeled in the SDM: occult (fog) deposition and dry (stomatal) deposition. We assume the SO_4_-S concentration in fog precipitation proportional to the SO_2_ concentration in the air. The amount of daily fog deposition (mg/m^2^) is calculated as the product of fog precipitation (mm), the average daily SO_2_-S air concentration (mg/m^3^), multiplied with a specific enrichment factor.

2 $${\textit{occult}} = {\textit{SO}}_{2}{\textit{Sf}}_{\textrm{occult}} {\textit{FOG}}$$

The determination of stomatal deposition also utilizes data of the SO_2_-S air concentration. As proxy for the degree of stomatal openness we use the soil moisture-dependent stress coefficient (*Kc*_s,tree_) (compare Dolschak et al. 2019). A value of one would correspond to fully opened stomata, whereas a value of zero would result in a complete shutdown of the stomatal gas exchange. The stands’ relative leaf area (*Kc*_LAI_) serves as proxy for the foliage surface (or the relative amount of stomata), exposed to sulfur dioxide. The function is stated as a product of the SO_2_-S air concentration (mg/m^3^), a specific enrichment factor, the stress coefficient, the relative leaf area and the daily mean wind speed (m/s), measured 2 m above ground.

3 $${\textit{dry}} = {\textit{SO}}_{2} {\textit{Sf}}_{{\rm dry}} {\textit{Kc}}_{{s,{{\rm tree}}}} {\textit{Kc}}_{{\rm LAI}} u_{2}$$

Belowground and biotic S fluxes and transformations are stated as a system of five ordinary differential equations which are solved simultaneously using the 4th-order explicit Runge–Kutta scheme. The temporal dynamics of SO_4_-S (*Sol*) in the soil solution are formulated, using the following equation. Infiltration (*Inf*) and the leaching (*Leac*) of sulfate-S are fluxes, driven by the WBM. *M*_stable_ and *M*_labile_ state the input to the soil solution via mineralization of the stabile organic and labile organic sulfur pool, respectively. *U*_plant_ and *U*_micro_ state the uptake of S by plants and soil microbes, respectively. *Ads* accounts for the adsorbed amount of SO_4_-S.

4 $$\frac{\textit{dSol}}{{{\textit{d}}t}} = \left( {{\textit{Inf}} + M_{{\rm stable}} + M_{{\rm labile}} } \right) - \left( {{\textit{Leac}} + U_{{\rm plant}} + U_{{\rm micro}} + \frac{\textit{dAds}}{{{\textit{d}}t}}} \right)$$

Foliage S (*Fol*) dynamics are described as the difference of plant uptake, multiplied with a constant fraction, describing the partition of plant uptake, routed to the foliage, plus dry deposition input to the canopy, minus the autumnal litterfall loss of S. The simulator neglects the possibility of S washout from the canopy. The seasonal foliage S pool is the only explicitly defined biomass pool in the SDM. As our interest does not lie in tree growth, we do not assume a change in S storage during stand development. It could be claimed that living biomass S storage is incorporated in the labile as well as the stabile soil organic S pool.

5 $$\frac{\textit{dFol}}{{{\textit{d}}t}} = f_{{\rm fol}} U_{{\rm plant}} + {\textit{dry}} - {\textit{litter}}$$

In our model, two pools are used to describe the dynamics of organic soil sulfur: A stable pool accounts for recalcitrant organosulfur compounds, and a labile pool accounts for more transitory organic sulfur species. The first pool could be viewed as corresponding to carbon-bond S, and the second could be assigned to ester-bond sulfate (McGill and Cole 1981). As a simplification, we assume that microbial S immobilization is only contributing to the labile pool. The balance of the labile organosulfur pool can be stated as the difference of the labile partition of plant uptake, which is not routed to the foliage, and litterfall, plus the microbial S assimilation, and the loss via mineralization. As a simplification, we assume a steady-state forest with an unchanging S content in the living woody biomass.

6 $$ \frac{\textit{dLabile}}{{{\textit{d}}t}} = f_{{\rm labile}} \left( {\left( {1 - f_{{\rm fol}} } \right)U_{{\rm plant}} + {\textit{litter}}} \right) + U_{{\rm micro}} - M_{{\rm labile}}$$

The stable pool is formulated analog to the labile pool. The stabile fraction of plant uptake and litterfall is formulated as 1−*f*_labile_.

7 $$ \frac{\textit{dStable}}{{{\textit{d}}t}} = \left( {1 - f_{{\rm labile}} } \right)\left( {\left( {1 - f_{{\rm fol}} } \right)U_{{\rm plant}} + {\textit{litter}}} \right) - M_{{\rm stable}}$$

Sulfate adsorption is modeled using the Langmuir isotherm. *M*_ads_ and *K*_ads_ are site-specific model parameters, and *Conc* states the SO_4_-S concentration in the soil solution. To express the kinetics of the process, it is coupled with a first-order decay function.

8 $$ \frac{\textit{dAds}}{{{\textit{d}}t}} = \lambda_{{\rm ads}} \left( {\frac{{M_{{\rm ads}} K_{{\rm ads}} {\textit{Conc}}\left[ {{\textit{mg}}/{\textit{L}}} \right]}}{{1 + K_{{\rm ads}} {\textit{Conc}}\left[ {{\textit{mg}}/{\textit{L}}} \right]}} - {\textit{Ads}}} \right)$$

The concentration of SO_4_-S in the soil solution is calculated as the storage in soil solution, divided by the product of soil depth and soil moisture.

9 $${\textit{Conc}}\left[ {{\textit{mg}}/{\textit{L}}} \right] = \frac{{{\textit{Sol}}\left[ {{\textit{mg}}/{\textit{m}}^{2} } \right]}}{{z_{\rm r} \theta }}$$

We derive the lambda value from the process’ half-life.

10 $$\lambda_{{\rm ads}} = - \frac{\ln 0.5}{{HL_{{\rm ads}} }}$$

The function stated below describes the response (*resp*_Q10_) of microbial S transformations to soil temperature. We use a base temperature of 8 °C that should resemble the annual average soil temperature at our investigated stands. The factor *f*_Q10_ describes the relative increase in the response when soil temperature rises by 10 °C.

11 $${\textit{resp}}_{\rm Q10} = e^{{\frac{{\ln f_{\rm Q10} }}{10}\left( {T_{{\rm soil}} - 8^\circ {\textit{C}}} \right)}}$$

In the WBM, the autumnal decrease in the LAI inside the interval *DoY*_LC_–*DoY*_LE_ (compare Dolschak et al. 2019) is described using the Smoothstep function (see Appendix). To define the relative amount of sulfur in the litterfall (litter) we first calculate the relative amount of daily litterfall (*f*_litter_).

12 $$ f_{{\rm litter}} = \left\{ {\begin{array}{*{20}l} {0,} \hfill & {{\textit{DoY}} < {\textit{DoY}}_{{\rm LC}} } \hfill \\ {1 - \frac{{{\textit{LAI}}_{{{\text{Kc}},{\text{DoY}}}} }}{{{\textit{LAI}}_{{{\text{Kc}},{\text{DoY}} - 1}} }},} \hfill & {{\textit{DoY}}_{{\rm LC}} \le {\textit{DoY}} < {\textit{DoY}}_{{\rm LE}} } \hfill \\  {1,} \hfill & {{\textit{DoY}} = {\textit{DoY}}_{{\rm LE}} } \hfill \\ \end{array} } \right.$$

To calculate the amount of sulfur in the litterfall biomass we multiply this value with the sulfur content in the foliage pool.

13 $${\textit{litter}} = f_{{\rm litter}} {\textit{fol}}$$

The latter function describes the response (*resp*_*θ*_) of microbial S transformations to soil moisture (see Sverdrup et al. 2007). At soil moisture levels below (*θ*_0_) and above a certain threshold (*θ*_3_), we claim that all microbial transformations come to a halt (*resp*_opt_ = 0). We assume optimal conditions (*resp*_opt_) at medium soil moisture between *θ*_1_ and *θ*_2_. In between *θ*_0_ and *θ*_1_ and accordingly in between *θ*_moist_ and *θ*_drown_ we assume linear transitions.

14 $${\textit{resp}}_{\uptheta } = {\textit{resp}}_{\hbox{min} } + \left( {{\textit{resp}}_{{\rm opt}} - {\textit{resp}}_{\hbox{min} } } \right)\left\{ {\begin{array}{*{20}l} {0,} \hfill & {\theta \le \theta_{0} \vee \theta < \theta_{3} } \hfill \\ {\frac{{\theta - T_{0} }}{{T_{1} - T_{0} }},} \hfill & {\theta_{0} < \theta \le \theta_{1} } \hfill \\  {1,} \hfill & {\theta_{1} < \theta \le \theta_{2} } \hfill \\ {1 - \frac{{\theta - T_{2} }}{{T_{3} - T_{2} }},} \hfill & {\theta_{2} < \theta \le \theta_{3} } \hfill \\ \end{array} } \right.$$

15 $$V_{m} = \frac{{V_{\hbox{max} } {\textit{Conc}}\left[ {{\textit{mg}}/{\textit{L}}} \right]}}{{K_{\rm m} + {\textit{Conc}}\left[ {{\textit{mg}}/{\textit{L}}} \right]}}$$

We describe microbial S immobilization using the Michaelis–Menten equation.

16 $$ V_{{{\text{m}},{\text{immobil}}}} = \frac{{V_{{{ \hbox{max} },{\text{immobil}}}} {\textit{Conc}}\left[ {{\textit{mg}}/{\textit{L}}} \right]}}{{K_{{{\text{m}},{\text{immobil}}}} + {\textit{Conc}}\left[ {{\textit{mg}}/{\text{L}}} \right]}}$$

The maximal assimilation rate depends on soil temperature and soil moisture.

17 $$ V_{{\hbox{max} ,{\text{immobil}}}} = V_{{{\text{coeff}},{\text{immobil}}}} {\textit{resp}}_{{\rm Q10,{\text{immobil}}}} {\textit{resp}}_{{\uptheta ,{\text{immobil}}}}$$

We describe the mineralization of stable organic S as first-order decay process, dependent on soil temperature and soil moisture.

18 $$ M_{{\rm stable}} = {\textit{Stable}}\lambda_{{\rm stable}} {{\textit{resp}}}_{{\rm Q10,{\text{mineral}}}} {\textit{resp}}_{{\uptheta ,{\text{mineral}}}}$$

The release of labile organic S (ester-bond) is also described as first-order decay process dependent on soil temperature and soil moisture. In addition, the rate of mineralization is also dependent on the SO_4_-S soil solution concentration.

19 $$ M_{{\rm labile}} = {\textit{Labile}}\lambda_{{\rm sulfatase}} {{\textit{resp}}}_{{\rm Q10,{{\rm mineral}}}} {\textit{resp}}_{{\uptheta ,{\text{mineral}}}}$$

The simulator comprises the effect of microbial sulfatase release on the kinetics of the labile organic S. A low SO_4_ concentration in the soil solution promotes the microbial release of sulfatase, thus accelerating the mineralization release of ester-bond sulfate (Scherer 2009). This is implemented by making the process’ half-life linearly dependent on the soil solution concentration. To set the linear dependence, half-lives are defined for 2 key solution concentrations (0 and 10 mg SO_4_-S/L).

20 $$ k_{{\rm sulfatase}} = \frac{{{\textit{HL}}_{{{\text{sulfatase}}.10}} - {\textit{HL}}_{{{\text{sulfatase}}.0}} }}{10}$$

The linear equation takes the form:

21 $$ {\textit{HL}}_{{\rm sulfatase}} = k_{{\rm sulfatase}} C\left[ {{\textit{mg}}/{\textit{L}}} \right] + {\textit{HL}}_{{{\text{sulfatase}}.0}}$$

The half-life is used to calculate the lambda value of the first-order decay process.

22 $$ \lambda_{{\rm sulfatase}} = - \frac{\ln 0.5}{{{\textit{HL}}_{{\rm sulfatase}} }}$$

The S uptake of deciduous trees is mediated through two distinct active carrier systems (Herschbach and Rennenberg 2001). Kreuzwieser and Rennenberg (1998) postulate high affinity and low affinity uptake systems. Low sulfate level in the aqueous phase of forest soils points to the dominance of the high affinity system (Herschbach and Rennenberg 2001). We simulate the uptake of S beech trees using Michaelis–Menten kinetics. We assume that plant uptake is only taking place during the growing season.

23 $$ U_{{\rm plant}} = \left\{ {\begin{array}{*{20}l} {\frac{{V_{{{ \hbox{max} },{\text{plant}}}} {\textit{Conc}}\left[ {{\textit{mg}}/{\textit{L}}} \right]}}{{K_{{{\text{m}},{\text{plant}}}} + {\textit{Conc}}\left[ {{\textit{mg}}/{\textit{L}}} \right]}},} \hfill & {{\textit{Kc}}_{{\rm LAI}} > 0} \hfill \\ {0,} \hfill & {{\textit{Kc}}_{{\rm LAI}} = 0} \hfill \\ \end{array} } \right.$$

We also claim that plants only take up a certain amount of S during the season. After a certain demand is met (*ACU*_starve_), plants start to throttle the uptake. After a second threshold is met (*ACU*_sat_), plants shut down the uptake of S completely. We calculate the accumulated uptake on a Day of Year base.

24 $$ {\textit{ACU}}_{{\rm DoY}} = \left\{ {\begin{array}{*{20}l}  {0,} \hfill & {{\textit{DoY}} = 1} \hfill \\ {U_{{\rm plant}} + ACC_{{{\text{DoY}} - 1}} ,} \hfill & {{\textit{DoY}} > 1} \hfill \\ \end{array} } \right.$$

The transition of the demand is modeled under the usage of the Smoothstep function (see Appendix).

25 $$ V_{{{ \hbox{max} },{\text{plant}}}} = {\textit{smooth}}\left( {{\textit{ACU}}_{{\rm DoY}} \left[ {{\textit{g}}/{\textit{m}}^{2} } \right],\;{\textit{ACU}}_{{\rm starve}} ,\;{\textit{ACU}}_{{\rm sat}} ,\;V_{{{\text{starve}},{\text{plant}}}} ,\;V_{{{\text{sat}},{\text{plant}}}} } \right)$$

### Model application

Before running the SDM, we define three target criteria, which mark a successful simulation:

- To bootstrap the SDM, we run a model spin-up, starting in the year 1770. As a primary requisite, the model must achieve steady-state conditions before atmospheric deposition starts to rise in 1880.
- The plots were sampled and analyzed in 2010, and the total S stock in forest floor and mineral soil (0–50 cm) was determined (E: 78.0 g/m^2^, J: 102.6 g/m^2^). The sum of all modeled soil S pools (adsorbed, stable organic, labile organic, soil solution) in 2010 has to be in a close range to the measured value.
- The simulator has to deliver a satisfactory reproduction of the observed timeline of the SO_4_-S concentration in the monitored soil solution.

We define plausible ranges for our set of 25 parameters (Table 1). The SDM was calibrated via simulated annealing (Kirkpatrick 1984). Performance criterion was the Nash–Sutcliffe model efficiency (*NSE*; Nash and Sutcliffe 1970), a function, which is generally used to evaluate hydrologic models.

**Table 1:** Parameter optimization results for the investigated sites

Abbrev.	Parameter and description	Unit	Exelberg	Jubiläum.
*f*_occult_	Fog (occult deposition) enrichment factor		33922.35	15395.36
*f*_dry_	Stomatal (dry) deposition factor		0.00	0.00
*Θ*_0,mineral_	Mineralization, lower threshold *Θ*	LL^−1^	0.07	0.01
*Θ*_1,mineral_	Mineralization, lower optimal *Θ*	LL^−1^	0.13	0.25
*Θ*_2,mineral_	Mineralization, upper optimal *Θ*	LL^−1^	0.23	0.33
*Θ*_3,mineral_	Mineralization, upper threshold *Θ*	LL^−1^	0.36	0.39
*Θ*_0,immobil_	Immobilization, lower threshold *Θ*	LL^−1^	0.04	0.04
*Θ*_1,immobil_	Immobilization, lower optimal *Θ*	LL^−1^	0.07	0.18
*Θ*_2,immobil_	Immobilization, upper optimal *Θ*	LL^−1^	0.16	0.29
*Θ*_3,immobil_	Immobilization, upper threshold *Θ*	LL^−1^	0.33	0.38
*HL*_stable_	Half-life, stable soil-organosulfur, opt. Conditions	Years	54.71	67.34
*HL*_labile_	Half-Life, Labile Soil-Organosulfur, opt. conditions	Days	21.99	13.00
*K*_m,immobil_	Immobilization, *K*_m_ parameter		1.81	1.05
*V*_m,immobil_	Immobilization, maximum rate, opt. conditions	SO_4_-S mg m^−2^	2.11	2.11
*f*_Q10,immobil_	Immobilization, Q10 Factor		2.37	2.77
*f*_Q10,mineral_	Mineralization, Q10 Factor		2.19	2.22
*V*_m,starve_	Plant Uptake, Maximum Rate at S Starvation	SO_4_-S mg m^−2^ d^−1^	0.11	0.07
*V*_m,sat_	Plant Uptake, Maximum Rate at S Saturation	SO_4_-S mg m^−2^ d^−1^	0.00	0.00
*K*_m,plant_	Plant uptake, *K*m parameter		1.16	0.95
TC_starve_	Plant uptake, threshold uptake, starvation	SO_4_-S mg m^−2^y^−1^	1141.79	913.76
*TC*_sat_	Plant uptake, threshold uptake, saturation	SO_4_-S mg m^−2^y^−1^	1367.79	2166.95
*f*_labile_	Plant uptake, routed to labile soil-organosulfur		0.18	0.07
*f*_fol_	Plant uptake, routed to foliage pool		0.74	0.82
*HL*_ads_	Adsorption, half-life	Days	205.00	71.10
*K*_L,ads_	Adsorption, half-saturation concentration	SO_4_-S mg L^−1^	12.53	11.68
*K*_max,ads_	Adsorption, maximum charge	SO_4_-S mg m^−2^	6085.48	7057.02
	Initialization 1770, labile soil-organosulfur	SO_4_-S mg m^−2^	5054.55	6804.58
	Initialization 1770, stable soil-organosulfur	SO_4_-S mg m^−2^	51175.13	61845.05
	Plant uptake 2010	SO_4_-S mg m^−2^	1345.80	1245.00
	Litterfall 2010	SO_4_-S mg m^−2^	992.40	1017.40
*NSE*	Nash–sutcliffe index		0.78	0.90
*RMSE*	Root-Mean-Square Error		0.44	0.28

The code file of the model (written in Base SAS 9.4) is available after request to the authors. The file contains the code for all model functions which are utilized in the Water Balance Model (WBM) and the Sulfur Dynamics Model (SDM). The recommended citation is: *this publication: code file of the Water Balance Model (WBM) and the Sulfur Dynamics Model (SDM).*

## Results and discussion

### Evaluation

**Fig. 4:**
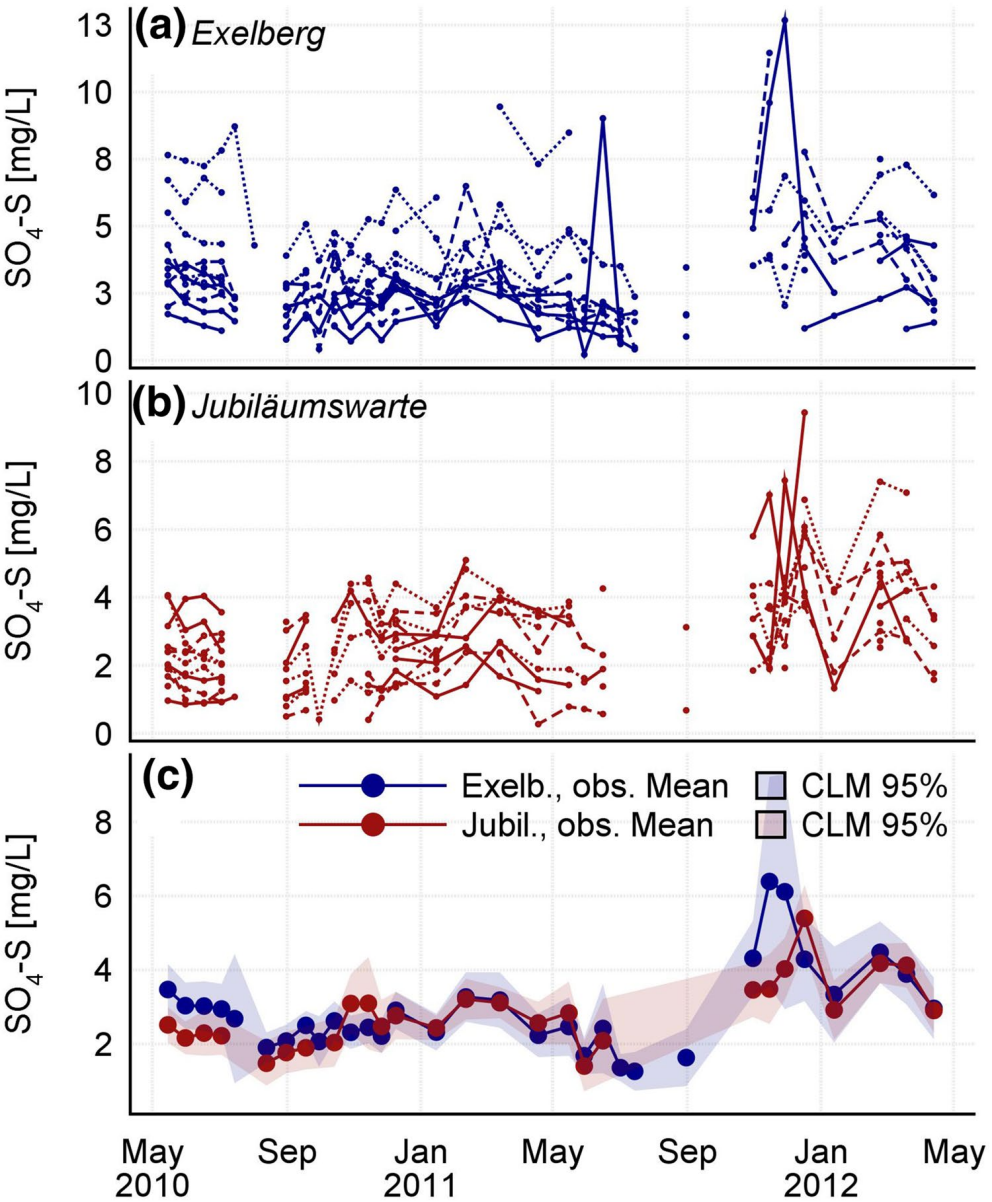
**a**, **b** Time series of SO_4_-S concentration, observed in soil solution. Each line represents one lysimeter. Eighteen lysimeters were installed per plot (6 repetitions and 3 depth classes). **c** To overcome the noise in the data, the SDM was fitted to site-wise mean concentrations. After calculating the mean, both sites reveal a very similar pattern regarding the temporal development of the soil solution concentration. This is leading to the assumption that soil S dynamics are controlled by over-regional drivers

Soil solution chemistry was monitored in a biweekly interval, on each site in 15 replications (Fig. 4a, b). This enables the calculation of robust site wise mean SO_4_-S soil solution concentrations per sampled event. The resulting timelines of both forest stands reveal a very similar pattern (Fig. 4c). The observed correlation gives a hint that sulfate solution chemistry might be driven by the same processes, on both stands.

**Fig. 5:**
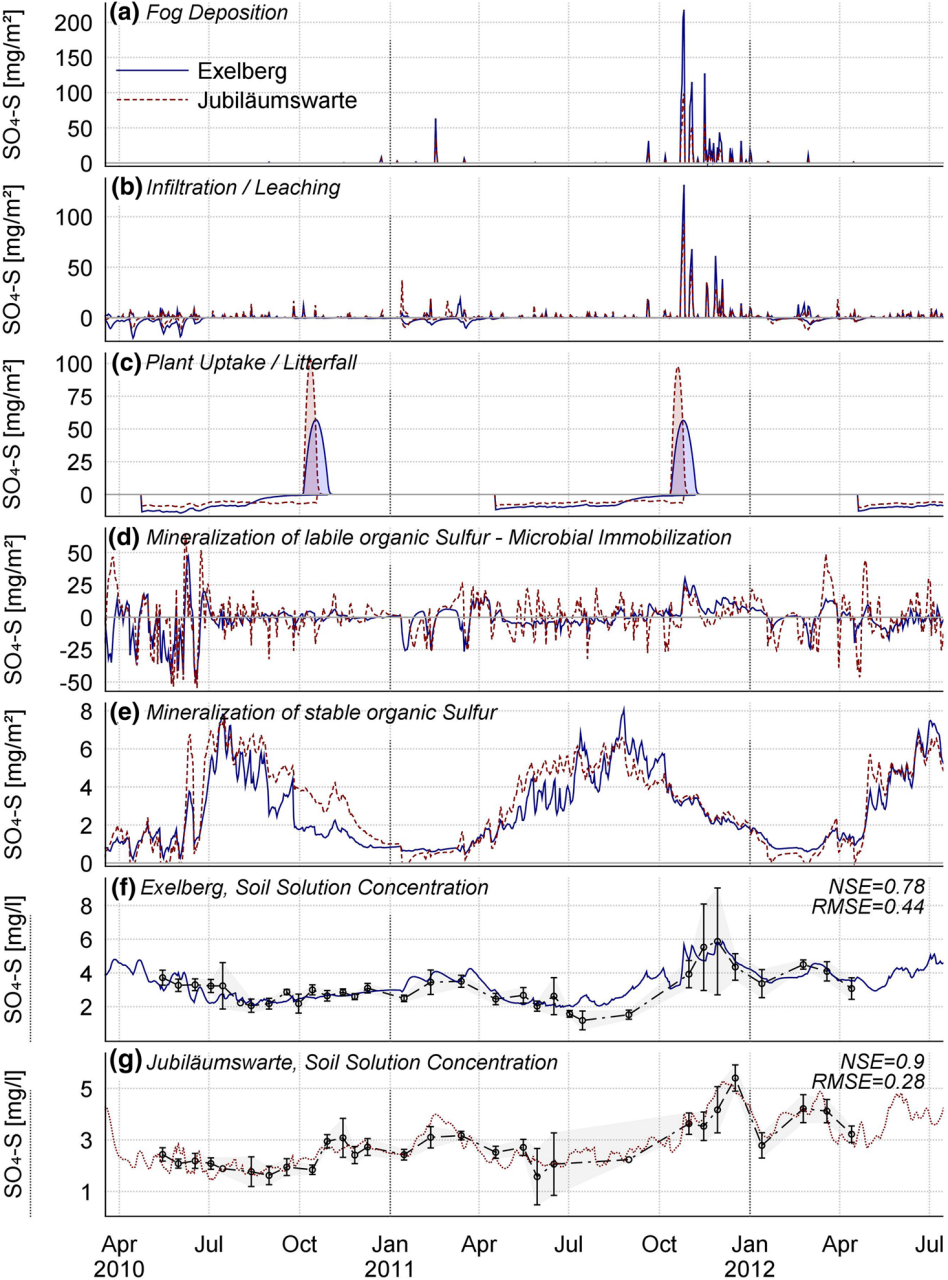
S fluxes during two years of field campaign. **a** Both stands experience a prolonged fog event in the autumn of 2011. During this period, we measured occult deposition loads between 200 and 500 mg SO_4_-S/m^2^. The simulation outcome suggests much higher values: From mid-October to the end of December 2011, the simulator predicts occult deposition sums of 1800 and 900 mg SO_4_-S/m^2^, for the Exelberg and Jubiläumswarte site, respectively. **b** During the field campaign, atmospheric inputs were dominated by the occult deposition event in the fall of 2011. Due to the coupling with percolation, leaching of SO_4_-S only takes place, when soil moisture is above water-holding capacity. This happens predominantly during the dormant season when evapotranspiration water fluxes are low. **c** We model plant uptake taking place only during the growing season. Because plant uptake is driven by their demand in our simulation, highest rates of S assimilation are achieved relatively early in the growing season. As the annual demand is met in late summer, plants start to throttle the uptake of S. During autumnal litterfall, the S, which is stored in leaf biomass, is transferred to the soil organic pool. **d** In this graph, we display the difference of mineralization of labile organosulfur and microbial immobilization. Positive values represent inputs to the soil solution meaning that mineralization is dominating. The erratic pattern is caused by shifted *Θ* optima for both processes (comp. Figs. 7, 8). Mineralization slightly favors higher *Θ* values than immobilization. **e** Mineralization is driven by substrate availability, *Θ*, and soil temperature. Maximal values are achieved at moderate *Θ* and high soil temperature (comp. Figs. 7, 8). **f**, **g** Observed and modeled timeline of the SO_4_-S concentration in the soil solution. The dash-dotted line illustrates the stands mean concentration at given time, and the error bars display the 95% confidence limit for the mean. The calibration led to sufficiently good results for both investigated forest stands. Both sites exhibit a peak in the solution concentration in late autumn of 2011. This is assignable to (1) high input loads via fog deposition and (2) mineralization excess during this period

**Fig. 6:**
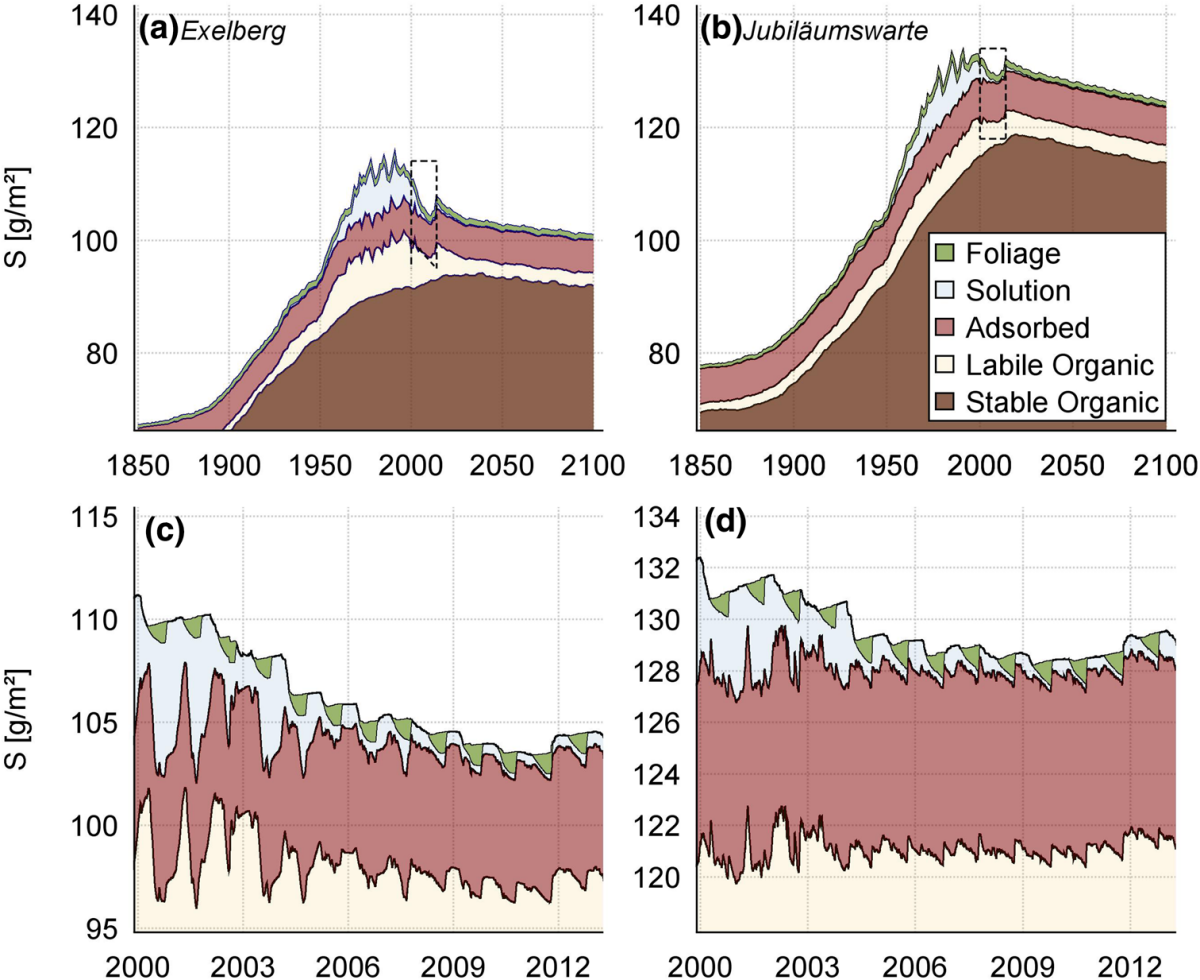
Temporal development of S pools over the whole model timeframe (a, b) and focused on the recent past (c, d). **a**, **b** The model displays a rise in the stands’ overall S stock, which is mainly assignable to the stable organosulfur pool (dark brown). Interestingly, this increase still has not tapered in the present decade. From the 1960s to the late 1980s S deposition peaked; high S loads are reflected in altered labile organosulfur and solute SO_4_-S in the soil. The amount of SO_4_-S, adsorbed in the soil, shows the lowest variability. Simulated sorption (E: 6.0 g, J 6.8 g) is during most periods close to the modeled maximum sorption capacity on both sites (*K*_max,ads_ = 6.1 g SO_4_-S m^−2^ Exelberg, 7.1 g SO4-S m^−2^ Jubiläumswarte). We estimated both stands’ total soil S pool in 2010. On the Exelberg site, an estimated amount of 78 g/m^2^ corresponds to a simulated soil S pool of 103 g/m^2^. The Jubiläumswarte soil S pool was estimated to 102 g/m^2^ in June 2010 and 128 g/m^2^ was simulated. Both sites exhibit two peaks of the total S pool: one at the end of the 1980s and one at the end of the monitoring period. The second peak might be caused by a high recurrence of deposition-rich fog events in this period. After 2020 both sites display a slow recession of S pools which does not reach steady state until the end of the prediction timeframe in 2100. **c**, **d** The model does not explicitly account for S, stored in non-green living plant biomass. The S uptake fraction, which is not assigned to foliage S, is routed to the soil organic S pool, where it is instantaneously exposed to mineralization. The simulated S content of foliage lies in the range of 1 g/m^2^. S is modeled, to be taken up steadily during the growing season. Autumnal litterfall leads to a sharp increase in the soil organic S pools

Regarding the timeline of SO_4_-S in soil solution, the SDM yields feasible results for both investigated stands: The simulations capture the observed timeline to a satisfactory degree (Exelberg: *NSE* = 0.78, Jubiläumswarte: *NSE* = 0.9, compare Fig. 5f, g, Table 1). The SDM overestimates the amount of total soil-S, which was quantified in 2010. For the E and J plot, the observed mean of the total soil-S pool down to a depth of 50 cm was 78.0 g/m^2^ and 102.6 g/m^2^ (Hanousek et al. 2017). The SDM delivers an estimate of 103 and 128 g/m^2^ (see Fig. 6c, d). Therefore, it overestimates both sites’ pools, each by approximately 25 g/m^2^. Given the fact that the presented model does not explicitly account for organic S, bond in living tree biomass other than foliage, one could argue that a portion of the modeled organic pool is contained in the aboveground plant tissue. For a beech-rich northern hardwood forest (Hubbard Brook Experimental Forest), Likens et al. (2002) give an estimate for the S, bound in aboveground living biomass, of 8.5 g/m^2^ roughly. Subtracting 1.0e g representing foliage S this narrows the gap, reducing the overestimation to approximately 17.5 g/m^2^.

Soil samples from both investigated sites were analyzed for S fractions by Hanousek et al. (2017). Additionally, they used material from a third site to create pooled samples. Adsorbed S was determined to a fraction of 6.4% of total soil S. The organic fraction accounted for 89%. Our modeling work delivers similar values. On both locations, adsorbed and organic fraction account for roughly 6.0% and 93.5% (Fig. 6c, d).

The organic fraction can further be divided into carbon-bonded S and ester-sulfates. For a nearby beech stand on similar bedrock the ester fraction was determined by Hanousek et al. (2017) via HI reduction (described in Kulhánek et al. 2011; Tabatabai 1996). The authors found that ester-sulfates account for approximately 38% of the entire organic fraction, which is in contradiction to Havlin et al. (2005) who suggest that ester-sulfates account for the majority of organic soil S. Our hypothesis that labile soil organosulfur corresponds to ester-sulfates must be rejected: Accounting for only 3.5% (E) and 4.5% (J) or organic soil S, our simulated labile pool (June 2010) is even smaller than reported by Hanousek et al. (2017).

For a beech stand on similar bedrock, Berger et al. (2009) reported an annual litterfall S flux of 0.45 g/m^2^. However, the S content in green foliage tissue was more than two times higher than in litter material, indicating translocation processes before leaf senescence. Our simulations exhibit values, of 1.0 g/m^2^ per year. This overestimation could be explained by the neglect of plant-internal S translocations in the current version of the SDM.

### Microbial S transformations

**Fig. 7:**
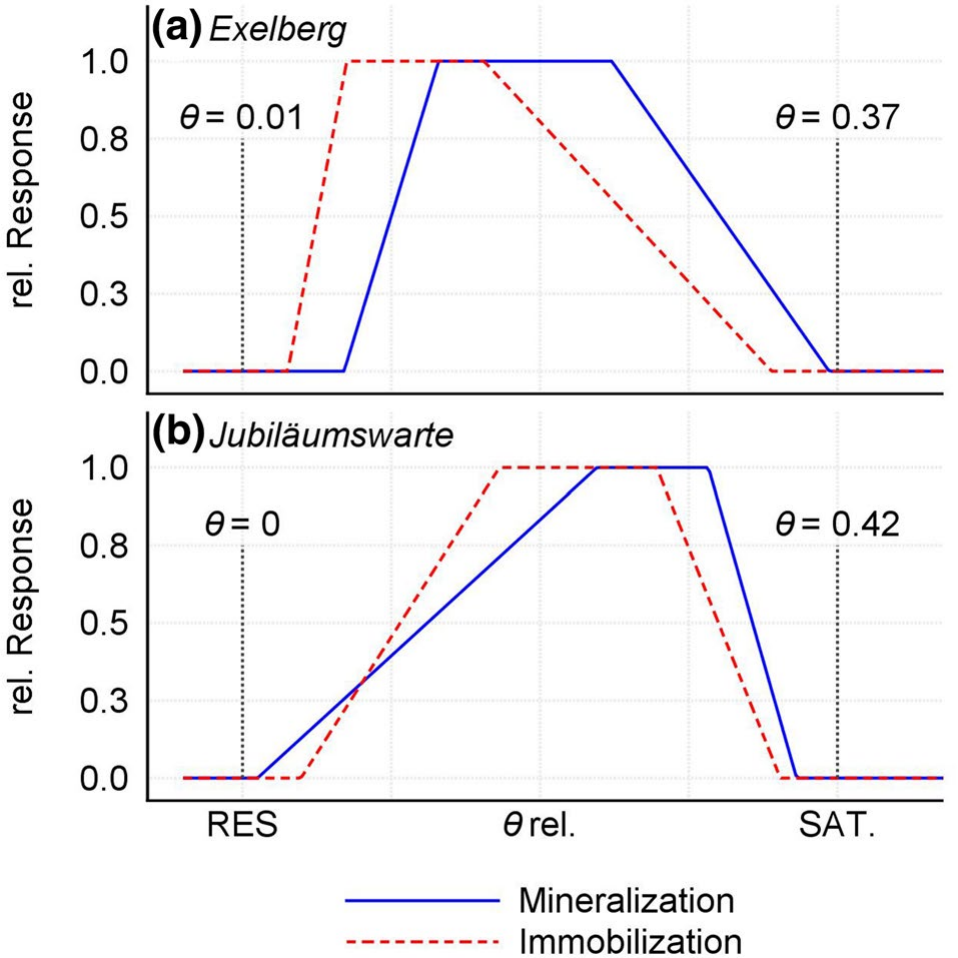
Response of microbial S transformations to soil moisture (*Θ*). *Θ* is displayed on a relative scale, whereas 0 corresponds to the residual water content and 1 to soil saturation, respectively. The response of microbial processes to *Θ* was described using a piecewise linear function. On both sites, the calibration led to mineralization and immobilization optima at medium *Θ* where a balanced water and oxygen supply are given. The range of maximal immobilization rates is slightly shifted to drier soil conditions (compare Table 1). In our model simulations, the non-congruence between the moisture response of immobilization and mineralization is responsible for short-term fluctuations in the SO_4_-S soil solution concentration

**Fig. 8:**
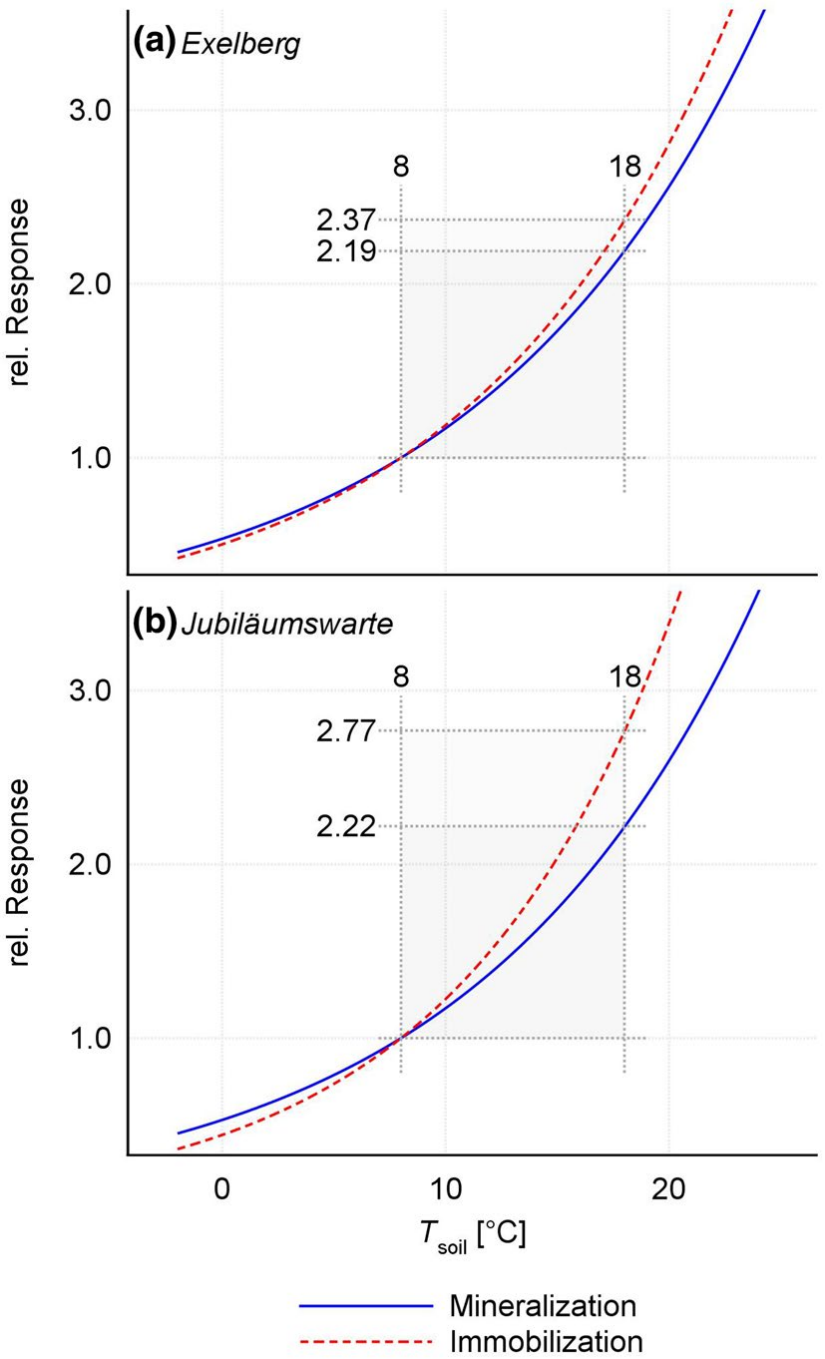
Response of microbial S transformations to soil temperature. The effect of soil temperature was modeled, using the *Q*_10_ temperature coefficient. We set the base temperature to 8 °C, which is close to the annual mean soil temperature of the sites. The calibration process delivered similar *Q*_10_ factors for both forest stands, whereas the temperature sensitivity of immobilization was slightly higher than the sensitivity for mineralization (compare Table 1)

Compared to other soil S pools, the microbial S pool is small. According to Chowdhury et al. (1999), microbially bond S accounts only for 1–4% of total soil S. Nevertheless, it is of great importance regarding plant nutrition due to its labile nature. Kertesz and Mirleau (2004) describe the contribution of microbial activity to the plant S supply: Plants take up S primarily in form of inorganic SO_4_ (Buchner et al. 2004). However, only a minor fraction of soil S is available in this form. As stated before, the majority is contained in organic material. Soil microbiota are responsible for the mineralization of organically bond S to inorganic sulfate. On the other hand, microorganisms are also driving the rapid immobilization of inorganic sulfate, first to relatively labile ester sulfates and furthermore, to more stable C-bonded S species (Ghani et al. 1993). In our formulation, S taken up by plants is routed to both stabile and labile organosulfur. In contrast to plant uptake, microbial immobilization contributes only to the labile pool. We describe mineralization and immobilization of the labile organic pool as temperature and moisture dependent. The soil moisture optima are slightly displaced (see Fig. 7): Immobilization is favored by dryer soil conditions. Immobilization also exhibits stronger response to warm conditions (compare Fig. 8). Kertesz and Mirleau (2004) see immobilization and mineralization as concurrent processes, occurring simultaneously in the soil. In our simulations, it is in fact the non-congruence of soil moisture and temperature response, which is driving a large portion of the observed fluctuations in the SO_4_ soil solution, favoring net mineralization under cool and moist conditions and net immobilization under warm and slightly dryer conditions.

### Occult deposition

**Fig. 9:**
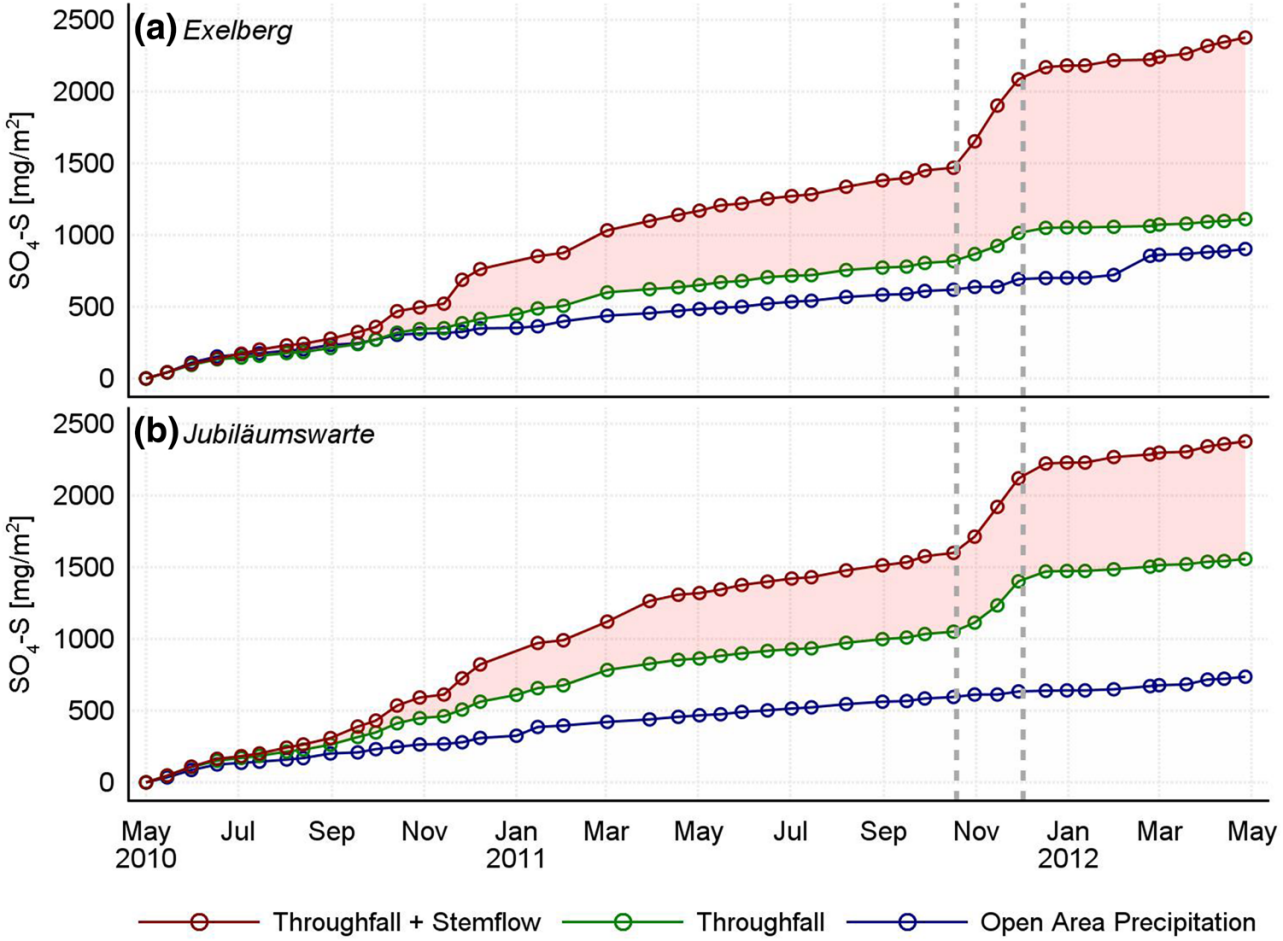
Accumulated SO_4_-S deposition on both investigated stands. During late autumn 2011 both sites experience a sharp increase in throughfall deposition. This period coincides with a long-lasting fog event; occult deposition seems to be an important sulfate source during such events

During two years of field campaign, we captured a prolonged fog event in late autumn of 2011. There is a solid body of work, asserting that fog water is enriched in sulfate, compared to rainwater (see Fowler et al. 1989; Lange et al. 2003). Especially in mountainous regions, fog can contribute significantly to the hydrologic and nutrient balance (Klemm and Wrzesinsky, 2007). We monitored deposition (1) in the open and (2) below the canopy (throughfall) and in form of (3) stemflow. The accumulated S deposition sums clearly display significant S inputs via occult deposition during the captured fog event (compare Fig. 9). The observed discrepancy between open area deposition and throughfall plus stemflow deposition indicates an extra input of approximately 500 mg SO_4_-S m^−2^. For our modeling purposes, we deal with open area deposition as input. The derivation of fog precipitation is presented in Dolschak et al. (2019).To scale from the mean daily aerial SO_2_-S concentration to the SO_4_-S concentration in the fog precipitation water, we apply an enrichment factor (see Lange et al. 2003). From 22nd October to 5th December 2011, the simulation yields an input of 1330 and 620 mg SO_4_-S m^−2^ in form of occult deposition for E and J. The spike in the soil solution at the end of 2011 (see Fig. 5a, f, g) is mostly assignable to fog deposition inputs. The observed and predicted high loads might be a result of the stands’ local conditions: In the Vienna Basin, winter fog events often coincide with southeasterly currents. When passing over the urban area, the air becomes enriched with pollutants. At the edge of the Vienna Woods it is forced to rise, condensation starts. The forest stands, which are located at the upper hill slope, facing south to east, are acting as a first barrier for the enriched fog, making them susceptible for the interception of high loads of atmospheric S. For a detailed description see Auer et al. (1989).

### Historic development and future prospect

**Fig. 10:**
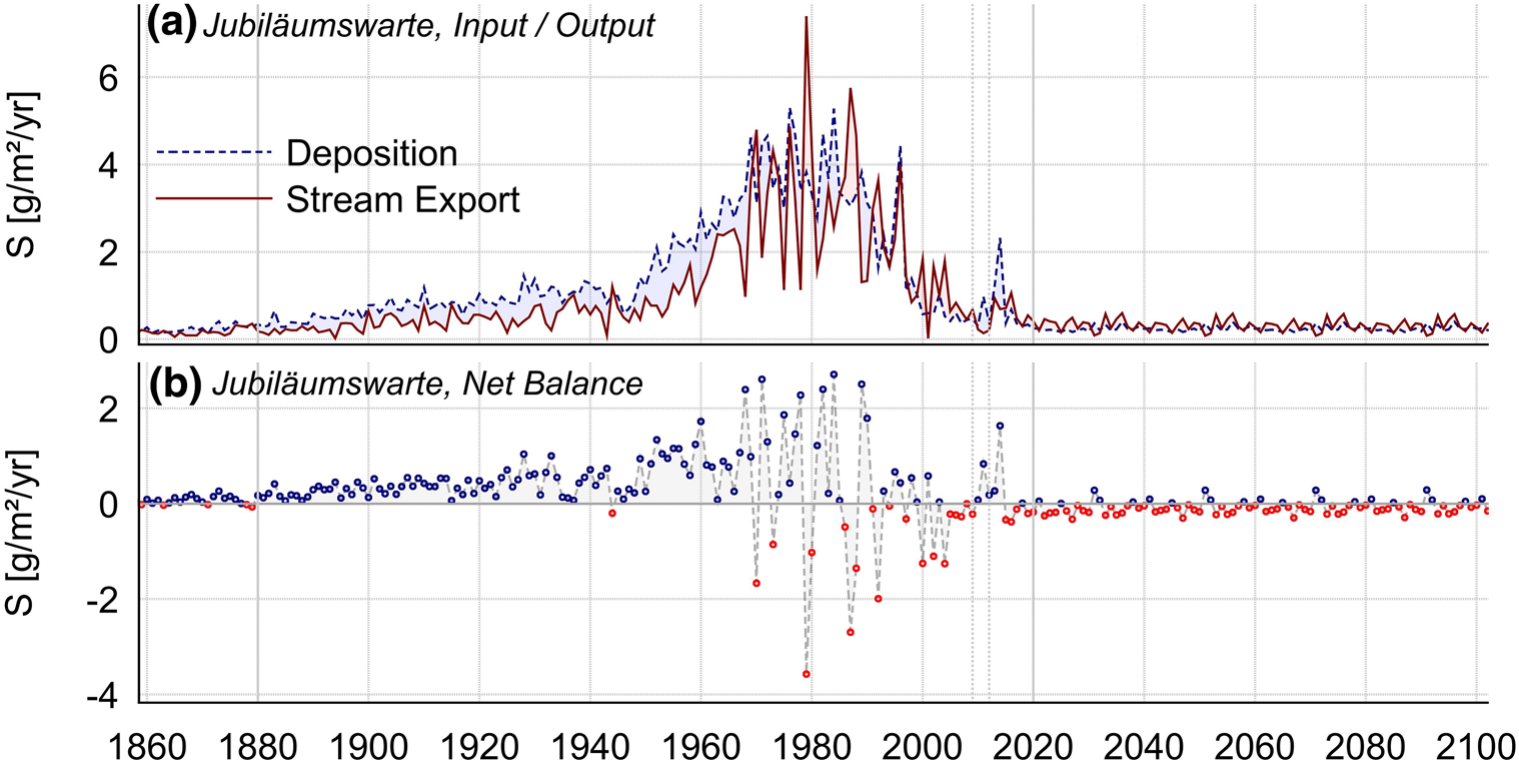
Annual S input versus annual output (I/O) for the Jubiläumswarte site. After the spin-up period I/O is in steady state. With rising atmospheric deposition, the system shifts to input excess in the end of the Nineteenth century. Highest inputs are modeled around the year 1980. In the late 1980s, the system shifts to output domination for the first time. A second smaller peak of input excess around the year 2015 is caused by a frequent occurrence of fog events in this period. After 2020 the model predicts a steady recession of input and output with output moderately dominating inputs for the remaining time period. At the end of the investigation timeframe in the year 2100, the system has still not fully restored I/O equilibrium and stream discharge of SO_4_-S remains slightly above the level of the pre-industrial era, ending in 1880

Both stands display a strong rise in the total soil S pool during the Twentieth century (compare Fig. 6a, b). This can be assigned largely to the accumulation of stable organosulfur originating from plant residuals. Interestingly, the accumulation of stable S has not fully tapered off in the present decade. In an attempt, to predict the future S output of two German catchments with the MAGIC model (Cosby et al. 2001). Prechtel et al. (2003) found that for one catchment the observed SO_4_ release could not be explained solely by adsorption/desorption processes in the soil. It was necessary to introduce an additional S source (968 mg m^−2^ yr^−1^) to match the simulated with the observed stream output. The authors suggested an S release from decomposition of organic soil constituents. In our work, S from mineralization of stable organosulfur adds a relatively steady annual amount between 700 and 1300 mg (1980–2020) to the budget. However, in our simulations, only a limited fraction of mineralized S enters the stream output. As the availability of other S sources might dwindle in the future, this fraction could further decrease. Much rather than being exported, it might be rapidly incorporated into living biomass and be cycled through the biosphere again. On the other hand, the remaining fraction which is leaving the system via seepage might hinder the achievement of a balanced input/output for many decades. Prechtel et al. (2003) point out that the investigated catchments could reach pre-industrial conditions in a few decades. They also claim that knowledge of origin and behavior of the postulated organic sulfur is crucial for the prediction of future trend in the stream chemistry. In our work, a large organic S pool with modeled half-lives up to 67 years will allow only a slow temporal recession of the catchment S export over the coming decades (see Fig. 10): Pre-industrial conditions in the stream discharge as well as a steady-state equilibrium are not reached over the whole investigation timeframe. The modeled amount of SO_4_-S, adsorbed in the soil, exhibits low variability over time. Simulated sorption is close to the modeled maximum sorption capacity on both sites (*K*_max,ads_ = 6.1 g SO_4_-S m^−2^ Exelberg, 7.1 g SO4-S m^−2^ Jubiläumswarte). On both sites, these pools remain almost constant over the entire model timeframe (see Fig. 6a, b) and therefore does not contribute to the present output excess of S.

Mineralization of S-containing soil constituents shows a strong response to soil moisture and soil temperature. Mitchell and Likens (2011) state the importance of the water balance in controlling the S output of forested watersheds: Soil moisture affects the net stream discharge, mineralization, desorption and weathering of S-bearing minerals. High temperatures might also accelerate processes. On the other hand, due to increase in evapotranspiration, high temperatures might lead to dryer soil conditions (see also Dolschak et al. 2019) impeding release and the export of S. Hence, how a changing climate might affect the stands’ S dynamics has yet to be explored.

### Inactive modules

#### Stomatal deposition

We assume that plants take up S primarily over the roots. Additionally, it is possible that airborne SO_2_ contributes to plant nutrition via the stomatal pathway (Rennenberg et al. 1990). We model stomatal deposition as a function of aerial SO_2_ concentration, the relative leaf area, the stomatal degree of openness (see Dolschak et al. 2019) and the wind speed. The calibration process always leads to stomatal deposition rates close to zero. It seems possible that (1), under current atmospheric SO_2_ concentration, stomatal uptake plays only a negligible role in plant nutrition or (2) the effect of stomatal uptake is comprised in other simulated modules.

#### Soil sulfatase activity

The SDM comprises the effect of microbial sulfatase release on the kinetics of the labile organic S. Deficits in the S supply (low SO_4_-S solution concentration) promote the microbial release of sulfatase, thus accelerating the mineralization of organically bond sulfate (Scherer 2009). We implement this by introducing half-lives of the labile organic pool, linearly dependent on the soil solution concentration. Yet during model fitting, concentration-dependent half-lives did not improve the performance of the simulator. Comparable to stomatal deposition, it seems possible that (1) other modeled processes already cover the effect of microbial sulfatase release. It might also be thinkable that (2) the observed SO_4_ supply in the soil solution is sufficiently high to fully meet the needs of soil microflora. Hence, the release of exo-enzymes might be initiated at solution concentrations much lower than observed.

## Conclusion

The objective of this work lies less in the long-term perspective of acidification recovery of forest soils; our primary interest lies in finding a mathematical description of the forest plant–soil system, capable to reproduce observed temporal patterns of the SO_4_-S in soil solution. Furthermore, we try to assign high-frequency fluctuations in the soil solution, observed on plot scale, to actual biogeochemical processes in the soil. After that, we venture a very cautious prognosis of the future development of the plant–soil system’s S budget on the investigated forest stands.

We identify microbial S transformations as important driver of the short-term dynamics in the soil solution. Yet on the long run, the mineralization of plant-originated soil organic S is responsible for the present input output imbalance. A large pool of historically accumulated organosulfur, which is decaying only very slowly, causes an output excess for decades or even centuries. Pre-industrial conditions in the stream discharge are not reached over the entire model timeframe. Besides that, occult deposition still contributes considerably to the supply of S to the forest.

For each plot, we deliver one set of parameters, which yields the best calibration result. We are aware that many different parameter configurations might lead to similar or even better outcomes. Nevertheless, the fact that it is even possible to find an adequate parameter set for each forest plot indicates that the current knowledge about forest soil S dynamics might be sufficient to describe the stands’ dynamics to a satisfactory degree. At least in the short period that was monitored no unexplainable artifacts remain, requiring fundamental reconsideration of the assumptions about the workings of soil S dynamics. At this point, it seems like we cover all essential processes, necessary to illustrate a comprehensive picture of the stands’ current S cycling.

## The code file of the model will be provided by the authors after request

*Filename*: CODE_WBM_SDM.sas.

*Title*: Code file (Base SAS 9.4) for the WBM and the SDM.

*Description*: The file contains the code for all model functions which are utilized in the Water Balance Model (WBM) and the Sulfur Dynamics Model (SDM).

*Recommended citation*: *this publication: code file of the Water Balance Model (WBM) and the Sulfur Dynamics Model (SDM).*

## Appendix

To achieve sigmoid shape transitions of a variables (*x*) response (*y*) inside a window (*x*_0_–*x*_1_), the Smoothstep function (compare Dolschak et al. 2015) was applied in several cases. *y*_0_ and *y*_1_ state left and right threshold responses, respectively. The variable *x* has to be normalized into an auxiliary variable (*t*) inside the interval 0–1.

26 $$t = \frac{{x - x_{0} }}{{x_{1} - x_{0} }}$$

The transition is described using a third-order polynomial.

27 $$ y = \left\{ {\begin{array}{*{20}l} {y_{0} ,} \hfill & {x \le x_{0} } \hfill \\ y_{0} + \left( {y_{1} - y_{0} } \right)\left( {3t^{2} - 2t^{3} } \right),   \hfill &  x_{0} < x < x_{1} \hfill \\  {y_{1} ,} \hfill & {x \ge x_{1} }  \hfill \\ \end{array} } \right.$$

In the article, the Smoothstep function is stated as:

28 $$ y = {\textit{smooth}}\left( {x,\;x_{0} ,\;x_{1} ,\;y_{0} ,\;y_{1} } \right)$$
